# Is the “Minimally Conscious State” Patient Minimally Self-Aware?

**DOI:** 10.3389/fpsyg.2020.539665

**Published:** 2020-11-12

**Authors:** Constantinos Picolas

**Affiliations:** ^1^Department of Philosophy, University of Patras, Patras, Greece; ^2^Department of Neurosurgery, Nicosia General Hospital, Strovolos, Cyprus

**Keywords:** self-awareness, minimally conscious state, vegetative state, pre-reflective self-awareness, experiential minimalism

## Abstract

Patients in a Minimally Conscious State (MCS) constitute a subgroup of awareness impaired patients who show minimal signs of awareness as opposed to patients in a Vegetative State who do not exhibit any such signs. While the empirical literature is rich in studies investigating either overt or covert signs of awareness in such patients the question of self-awareness has only scarcely been addressed. Even in the occasion where self-awareness is concerned, it is only higher-order or reflective self-awareness that is the target of such investigations. In the first part of this paper, I briefly review the relevant clinical neuroscience literature to demonstrate that the conception of self-awareness at play in such studies is indeed that of reflective self-awareness. In the second part, I present the philosophical notion of pre-reflective (or minimal) self-awareness. This is shown to primarily refer to the implicit awareness of our embodied subjectivity which essentially permeates all our experiences. As discussed, this minimal self-awareness is not specifically addressed when clinically or experimentally assessing patients in MCS. My suggestion is that neuroimaging studies targeting minimal self-awareness as in First-Person Perspective-taking paradigms could be used with MCS patients to shed light on the question of whether those individuals are minimally self-aware even in the case where they lack self-reflective abilities. Empirical evidence of this kind could have important theoretical implications for the discussion about the notion of self-awareness but also potential medical and social/legal implications for awareness impaired patients’ management.

## Introduction

According to a recent discussion in the area of Philosophy of Mind and Phenomenological Philosophy, our psychological states are characterized by an inherent self-awareness considered to be a constitutive part of our experiences. This implicit awareness of the self seems to be necessary for the very existence of subjective states in normal subjects (Strawson, 2009; Zahavi, 2017). It is often called *minimal* or *pre-reflective* self-awareness^1^ (PRSA) in the sense that it is not a higher-order or reflective grasp of the self. And in an apparent association to the above because of the similar terms involved, in clinical neuroscience, there is a seemingly relevant and thriving discussion about some pathological subjects being in a *Minimally* Conscious State (MCS) after recovering from severe brain damage.

In this paper, I intend to query about the following: Should we trace the difference between patients in MCS – who are minimally capable of awareness – and patients in Vegetative State (VS) – who are considered utterly unaware – back to the former’s exclusively possessing PRSA? My interest is to try to make sense of what is it that clinicians and neuroscientists mean (or should mean) when describing a patient in MCS as being minimally conscious and on the other hand to examine whether the neuroscientific literature on the topic can shed any light on the philosophical debate about self-awareness. To pursue this double interest I will first make a short presentation of the recent literature around the so-called disorders of consciousness (DOC) and then give a brief account of the philosophical notion of minimal self-awareness. I will conclude this paper with a discussion about the respective concepts of minimal awareness in these two fields and their potential interrelatedness.

## Disorders of Consciousness

According to a widely used medical definition “*[c]onsciousness is the state of full awareness of the self and one’s relationship to the environment*” and it “*has two major components: content and arousal*” (Posner et al., 2018: p. 5). That is, to be conscious requires one to be awake and while awake to have conscious contents. Conscious contents are accordingly distinguished into two broad categories, those that amount to the awareness of oneself and those that amount to the awareness of one’s environment. In clinical practice one is evaluating a patient’s *level* of consciousness with everyday practice diagnostic scales such as the Glasgow Coma Scale–GCS (Teasdale and Jennett, 1974) and her *content* of consciousness with targeted questions about awareness of self, place and time.

Now in clinical situations there are occasions where a patient is unresponsive and unable to wake up. In these cases, there is no demonstrable arousal state nor any expressed conscious contents and the patient is said to be in coma. But the case sometimes is that a patient is able to wake up but is utterly unresponsive. In this occasion he is said to be in a Vegetative State. The term was coined in 1972 by Jennet and Plum to refer to the state of some brain-damaged patients who after regaining their sleep-wake cycles from a period of being comatose, did not seem to show any signs of awareness either of themselves or their environment (Jennett and Plum, 1972). The term “vegetative” is of course not referring here to the autonomic part of the nervous system, responsible for basic life functions such as sleep-wake cycles, breathing, digestion, thermoregulation, etc. In fact, because of the pejorative connotation involved in the above notion the neutrally descriptive term *Unresponsive Wakefullness Syndrome* was also introduced recently (Laureys et al., 2010) and is currently used by an increasingly number of authors.

A few years later, The Multi-Society Task Force on Pvs (1994) (Persistent VS) defined the VS as the “*clinical condition of complete unawareness of the self and the environment, accompanied by sleep-wake cycles, with either complete or partial preservation […OF…] autonomic functions […AND…] no evidence of sustained, reproducible, purposeful, or voluntary behavioral responses[.]*” (1994: 1499). To better highlight this state as opposed to the case where subjects are indeed capable of awareness these patients are now characterized as not manifesting *voluntary behavior*. This voluntary behavior is the sign the examiner looks for as evidence of their being aware of themselves and their environment.

In subsequent years though, there were reports of behaviorally diagnosed patients in VS that did seem to show minimal signs of such voluntary behavior. So in 2002, the Aspen Neurobehavioral Conference Workgroup published a set of diagnostic criteria for MCS a disorder to be distinguished from VS by being “*a condition of severely altered consciousness in which minimal but definite behavioral evidence of self or environmental awareness is demonstrated*” (Giacino et al., 2002 p. 350–351). According to these criteria, a patient in MCS should be at least capable of minimally construed purposeful behavior. In 2004 the more refined Coma Recovery Scale-Revised (CRS-R) was published by the same team as a diagnostic tool to reliably discriminate MSC from VS patients by including more parameters than merely the two mentioned above (Giacino et al., 2004). This widely used scale was designed to help clinicians recognize overt responses to auditory and visual stimuli indicative of self and environmental awareness. It is designed, that is, to detect minimal signs of voluntary behavior and thus to enable the examiner to ascribe awareness (even if minimal) to the subject examined.

The CRS-R is a clinical tool, it is a means to observe the behavior of human beings and to infer from that behavior whether they are conscious or not. It is of no help in the potential scenario where one might be conscious but shows no behavioral signs of it. And indeed an article came in 2006 (Owen et al., 2006) demonstrating that this was just the case in a neuroimaging study where a woman fulfilling all the clinical criteria of VS showed fMRI activity identical to normal subjects when asked to imagine about exploring the rooms of her house and playing tennis. This and other (neuroimaging, electrophysiological) subsequent studies confirmed that a number of patients behaviorally diagnosed as being in VS were nevertheless capable of voluntarily performing imaginative tasks (Boly et al., 2007) or, in other cases, of demonstrating executive functions (Naci et al., 2014). One such patient was even shown to respond with specific imaginative tasks as (proxies of) yes/no responses to communicate with the examiners (through the related fMRI patterns) when asked personal questions (Monti et al., 2010).

## Self-Awareness in Clinical Neuroscience

Few points to notice from the above brief exposition are the following:

• A patient in VS is considered to be utterly unaware either overtly (that is, as indicated by his behavioral responses to stimuli) or covertly (as indicated by objective studies such as neuroimaging).
• A patient is considered to be Minimally Conscious on the other hand if he does show behavioral responses considered to be voluntary and the various clinical assessment tools are designed in such a manner as to pick up these responses.
• A totally unresponsive patient, one who shows no voluntary behavior is not to be considered Vegetative unless evidence of covert awareness is ruled out by objective means (neuroimaging, neurophysiological studies).

But what kind of concept of awareness does clinical neuroscience presuppose when such an awareness is demonstrated by clinicians and laboratory neuroscientists to (overtly or covertly) occur? As we saw, clinical neuroscientists have the notion of two broad kinds of awareness. Self-awareness and awareness of one’s environment. Awareness of one’s environment should be regarded here in quite broad terms involving not only the perceptual comprehension of the space around one, but also understanding of the potential practical use of objects, understanding of the actions of other subjects, mindreading their intentions, language comprehension etc. And according to the more narrow clinical diagnostic setting presented above, it is *voluntary* behavior, as this occurs in response to comprehension of aspects of the environment presented by sensory means (verbal commands, practical objects to be manipulated by hand), that is mostly considered indicative of awareness.

But what about self-awareness? Does the clinical neuroscience of DOCs as presented in the recent literature on the matter take into consideration this type of awareness? And if it does, what concept of it does it possess? Even if it doesn’t explicitly take into consideration self-awareness what concept of self-awareness does it implicitly presuppose?

This is the kind of questions I want to raise in this section in an attempt to force into relief the conception of self-awareness at play in DOC studies. In the next section I will juxtapose this to the notion of minimal self-awareness as presented in the contemporary philosophical literature on self-awareness.

I’ll begin by briefly discussing the JFK CRS-R clinical scale because it is widely used among clinicians and because it represents how clinicians expect awareness to manifest in a psychological subject. In the next subsections I will also briefly focus on neuroimaging and neurophysiological studies explicitly designed to present covert awareness.

## CRS-R

This behavioral diagnostic tool is divided into six parts each of which quantifies patient responses to different stimuli^2^.

• There is the Auditory Function Scale which monitors motor responses to sound. What is considered indicative of (minimal) awareness here is whether there is a reproducible motor response to verbal commands. This presupposes that the patient has regained the capacity to understand speech and is at least neurologically able to attempt an appropriate motor response.
• The Visual Function Scale monitors responses to visual information and what counts as evidence of awareness is the fixation of gaze or visual pursuit of the subjects’ own-face as seen by him in a mirror. Additionally reaching for seen objects and signs of object recognition is regarded as evidence of environmental awareness.
• In the Motor Function Scale, it is the localization to pain and object manipulation.
• In the [Oromotor] Verbal Function Scale it is intelligible verbalization as opposed to incoherent vocalizations.
• In the Communication Scale is evidence of attempted intentional communication (including also inappropriate non-functional attempts) and the last part of CRS-R detects the level of Arousal by evaluating whether the subject can be awakened and maintain his attention to sensory stimuli.

So according to this clinical scale, a patient with impaired consciousness is considered to be minimally conscious when consistently exhibiting appropriate behavioral responses to verbal commands, when demonstrating practical understanding of seen objects (affordances)^3^, when appearing to experience bodily pain (by reacting with an avoidance response), when he tries to communicate and when he shows recognition of his own-face in the mirror.

Importantly, self-awareness can be ascribed to this subject by his appropriate response to visual self-referential stimuli, in this case by showing signs of recognition of his own-face. I say this is important because it indicates that the conception of self-awareness at work in the MCS related clinical neuroscience literature is this type of higher-order, thematic self-recognition which is not the same as the philosophical conception of minimal or PRSA that we will tackle later^4^.

## Neuroimaging and Electrophysiological Studies

Current cortical functional anatomy models distinguish unimodal, primary information processing areas for each sensory modality from multimodal association areas where higher-order or conceptual information is processed. So the idea was that patients in VS who by definition possess no capacity for awareness would not show significant activation in the association areas (Boly et al., 2005). And that was the case initially until some VS diagnosed patients were found to present near-normal activation of the appropriate cortical regions, as we previously mentioned (page 2), following auditory commands to perform specific imaginary tasks (Owen et al., 2006; Cruse and Owen, 2010; Monti et al., 2010; Owen, 2013; Marino et al., 2017).

One objection raised to interpreting these results as evidence of awareness was that these patterns of brain activation might only be indicative of unconscious processing of auditory stimuli by higher-order regions (Greenberg, 2007; Nachev and Husain, 2007), functioning in this pathological case like mere passive “*islands of function*,” inadequate of supporting actual awareness. That is, the case might be that there is merely a disjoint activation of these brain areas which in the normal case are involved in voluntary imagination tasks in a functionally coherent and non-automatic manner. Responding to this objection the authors of the aforementioned study insisted that since “*the observed activity was not transient, but rather persisted for the full 30s of each imagery task*” these “t*emporally sustained [fMRI.] activations are impossible to explain in terms of automatic responses*” (2013: 118). And indeed it makes good sense to think that a temporally sustained activation that lasts for as long as the prompt to perform an imagery task specifies, and the subsequent change of the pattern of activation at the time the prompt for a new task is given, must be indicative of an alert and comprehending subject capable of having intentions and conscious attention shifting.^5^

So what counts as evidence of awareness in these studies is for the subject to perform a specific imagery act when specifically asked to, a type of voluntary act which resonates with the volitional component which the clinical scale focuses on. The content of the imagery act is a representation of a real-life motor act and the method of detecting this is indirect by neuroimaging.

Is this a case of addressing self-awareness? In the case where I imagine navigating through the rooms of my house or in imagining playing tennis or any similar action I presumably imagine *myself* acting as such and this presupposes a self embedded in the experience. But the psychological act of imagining that I perform a motor action does not specifically address this embedded self as, for instance, the case is with own-face recognition. The subject in this latter case *intends and mentally grasps* his own face *as his own*. Rather, the aforementioned fMRI imagery task addresses neural correlates of such phenomenological data as, say, the very ability of the subject to imagine an action and the action imagined (navigating a house, playing tennis) including the intentional objects involved (rooms/items of the house, playing field/racket/tennis ball/opponent etc.). It does not *specifically* address an intended attribute of the subject’s self. In this respect, the currently published neuroimaging studies about DOCs do not seem to directly address self-awareness.

But what about electrophysiological studies? Contrary to neuroimaging studies, self-awareness *has* been addressed in this occasion. For instance, in various Event-Related Potentials (ERP) studies the so-called P300 wave is detected when attention is grasped on auditory stimuli of interest (Laureys et al., 2007). And in a number of these studies, the subject’s own-name has been used as a self-referential stimulus either in passive paradigms where it is randomly mentioned or in active paradigms when the subject is asked to count the number of times her name is heard (Schnakers et al., 2008).

The presence of the P300 wave, in this last setting of self-referential stimulus presentation, is considered by the relevant literature as indicative of own-name recognition in healthy subjects. In DOC patients, though this conclusion is not that straightforward since a number of VS diagnosed patients also seem to present the P300 activation when presented with self-referential stimuli^6^. Even so, as with the case of *own-face* recognition in the clinical scale, this is similarly an experimental paradigm of an explicit grasping of a self-attribute as one’s own (passive own-name recognition or actively counting it) and not of one focusing on implicit self-awareness (which does not involve a reflective grasp of a specific self-trait, as we’ll see in the next section)^7^.

## Minimal Self-Awareness

We now move to a brief presentation of the concept of minimal self-awareness as it manifests in recent discussions in the philosophical literature of self-awareness. I cannot and do not intend to cover here the quite extensive modern literature on the matter of self-awareness from Shoemaker (1968) on. Rather I will focus on recent, phenomenology inspired, incarnations of this topic which focus on embodied subjectivity.

In a recent review article about self-awareness in patients with impaired consciousness by an influential research team, own-face and own-name recognition were presented to be the only types of self-referential stimuli used in functional studies with patients with DOC in agreement with our presentation above (Laureys et al., 2007). In their theoretical exposition of the notion of self-consciousness the authors distinguish “*six types of representation about self-awareness*” following distinctions previously made by Zeman (2005):

(1) Self-Consciousness as the embarrassment of myself in the presence of others, the colloquial use
(2) Self-Consciousness as “self-detection” in the sense of my bodily self-awareness in proprioception, interoception, and awareness of motor agency
(3) Self-consciousness as “self-monitoring,” the ability to reflectively grasp my-self as a practical agent in action recall and anticipatory motor planning
(4) Self-consciousness as “self-recognition,” as in mirror self-recognition
(5) Self-Consciousness as “awareness of awareness,” as the awareness of myself being a subject of beliefs and intentions, a “theory of mind” consciousness
(6) Self-Consciousness as awareness of myself as the hero of my personal narrative based in part to “*the capacity to relive our past in the form of ‘mental time-travel*”’ (Ibid: 5)

Let us focus on the 2nd type in Zeman’s taxonomy, that of bodily self-awareness. This type of self-awareness is the awareness of myself as the particular embodied subject of experience that I am (and each one of us is), it refers to the fact that I am at any moment of my conscious life *implicitly* aware of my body as the experiential center of my perceptual and practical engagement with the world. Bodily self-awareness is of an altogether different type from the other types mentioned by Zeman in that all of those require me to possess a self-*representation*. I need to have myself as an intentional object (to use the phenomenological term) or to reflectively represent myself as such in order to be able to monitor myself in time, to recognize a face in the mirror as my own or to speak about myself as a diachronic entity in a personal narrative. Contrary to all that, bodily self-awareness as PRSA does not require a representation of myself, rather it is a tacit awareness of myself as subject, the embodied subject of my first personal experience.

As Zahavi, a principal perpetuator of this view puts it, “pre-reflective self-consciousness *is precisely taken to differ from reflective self-consciousness by being an intrinsic non-objectifying form of self-acquaintance*” (2017: 4). Similarly, Gallagher distinguishes minimal selfhood from narrative selfhood. While the first term refers to “*a consciousness of oneself as an immediate subject of experience*,” the second term refers to the ability to speak of my life story in a thematic grasping of myself as a single individual persisting in long term time (Gallagher, 2000)^8^.

So according to this *experiential minimalist* view (Zahavi, 2017: p. 3), I am minimally self-aware in a pre-reflective manner in all aspects of my wakeful and conscious life. And this is the case even when I am not explicitly focusing on myself as when I do mentally grasp myself in cases where I self-reflect in episodic memory acts, anticipatory planning, personality or behavior self-judging or in thinking about my beliefs, desires, and intentions. PRSA (pre-reflective self-awareness) is thus always present if I am to be conscious at all. And importantly, whereas PRSA is a fundamental condition for the possibility of awareness in general, a person can be conscious without necessarily practicing self-reflecting. Opposing this view is the position that experiences are consciously mine only when they are reflectively grasped by a higher-order act (Rosenthal, 1986; Lycan, 1987; Van Gulick, 2001) but also the eliminativist position that experiences are not characterized by self-awareness at all and all that appears in experiences are the intentional objects of such experiences (Schear, 2009; Howell and Thompson, 2017).

We were inquiring about the self-awareness status of patients in MCS. We saw that when the relevant clinical neuroscience literature addresses this, it implies the possession of reflective or higher-order self-awareness in such patients. But in theoretical discussions about self-awareness a concept of a minimal or pre-reflective variety is more narrowly defined, a more fundamental type of self-awareness underscoring our reflective capacities themselves.

Now the question can be raised: How exactly does this minimal self-awareness notion relate to the understanding we have of the presence in patients in a MCS of “*minimal*” awareness? That is, how does the philosophical concept of minimal self-awareness relate to the clinical neuroscience concept of minimal awareness?

As we presented the case in the previous sections (pages 3–6) a patient in MCS in considered minimally aware because he shows minimal signs of overt or covert voluntary responses to verbal commands or other sensory stimuli as evidenced in CRS-R and neuroimaging studies. As for self-awareness is concerned, we saw that it is only higher-order self-awareness that this domain focuses on in the few cases that it does. So similarly, such a patient is considered minimally self-conscious when he presents minimal signs of this higher-order self-awareness. But surely, what does the term “minimal awareness” refer to here?

**FIGURE 1:**
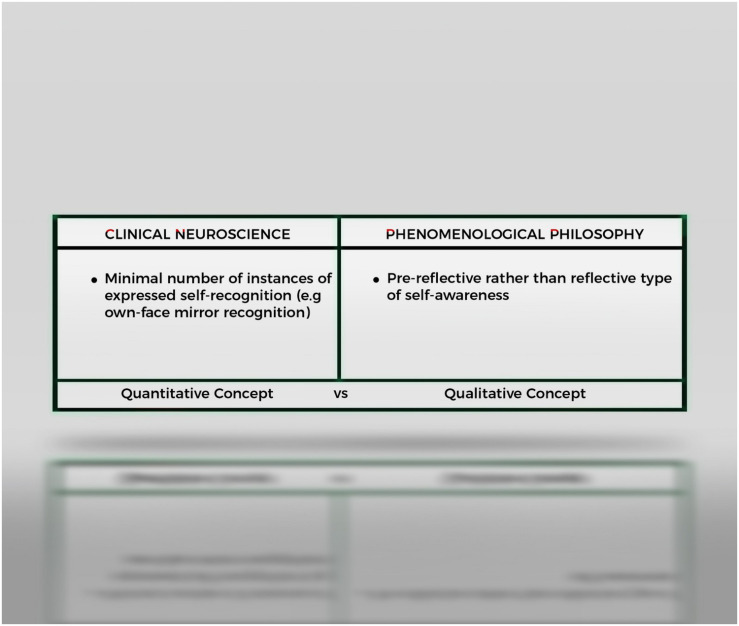
The concept of “minimality” has a different connotation in clinical neuroscience as opposed to that of the philosophy of self-awareness. For clinical neuroscience a subject is minimally self-aware in the sense that he expresses a minimal number of instances of such behavior. In phenomenological philosophy a subject is considered to be minimally self-aware because he is thought to possess, in addition to higher-order or reflective self-awareness a qualitatively different type of self-awareness, namely pre-reflective self-awareness.

I think we can observe that the clinical concept of *minimal* awareness is *quantitative* as it refers to the presence in a subject of a *minimum* number of voluntary behavior traits. And so is the clinical concept of minimal self-awareness. That is, it does not amount to a qualitative difference in self-awareness (e.g., pre-reflective vs. reflective self-awareness) but to a difference in the quantity of self-awareness traits overtly of covertly present in patients with DOC. In other words: According to clinical neuroscience, patients in MCS are *minimally aware* because they possess a minimal quantity of awareness traits (signs of voluntary behavior etc.) and they are *minimally self-aware* because they possess a minimal quantity of higher-order self-awareness (own-face, own-name recognition). Or, a patient in MCS is minimally self-aware because he manifests in *less* instances higher-order self-awareness traits than a normal subject does (Figure 1).

This interpretation is also compatible with the recent proposal in the DOC literature of a further differentiation of MCS into an MCS+ and MCS− type using some quantitative criteria. Accordingly any patient with any additional language-dependent behavior (command-following, intelligible verbalization, intentional communication) has less functional disability and should be characterized as being in MCS+ (Aubinet et al., 2018; Thibaut et al., 2020).

In light of the above thoughts, we can now return to our initial question (page 2): *Do patients in MCS, being minimally capable of awareness, differ from patients in VS because the first possess PRSA*? If we take this question to be asked from the aspect of clinical neuroscience and its current concept of self-awareness the answer is negative. From that aspect, a patient in MCS is minimally self-conscious because he possesses a *quantitatively* minimal capacity of higher-order self-grasping. The concept of PRSA is irrelevant (yet) to this research field.

But if, after clearing our view with our previous analysis, we consider this question anew it is actually a significant question to ask. Indeed, how does the situation stand with PRSA in awareness impaired patients? Is the patient in MCS minimally aware not only because he manifests a minimal number of awareness (and self-awareness) traits *but additionally* because they become pre-reflectively self-aware when emerging from coma or VS? How should we empirically proceed to explore such a scenario? And what would this potential patient’s possession of PRSA mean for theoretical approaches such as the experiential minimalist one?

For instance, consider the case where an awareness impaired person might be pre-reflectively self-aware without actually exhibiting any explicit (behavioral) or implicit (neuroimaging) signs of reflective self-awareness. This question is important both from a theoretical and an empirical point of view. It would be theoretically rewarding to gain empirical evidence from studies of MCS about the alleged presence of minimal self-awareness without the additional presence of any higher-order reflective capacities. This might give support to the view of the proponents of experiential minimalism that a reflective grasping of the self is not a necessary condition for awareness and also against the eliminativist view that no self-awareness figures necessarily in our experiences.

But on the other hand, perhaps PRSA is impossible without at least the potentiality for reflective self-awareness, that is, perhaps in us human beings, self-awareness is only possible when we have already developed our reflective capacities in early childhood. This could be the case even though we might maintain the position that a psychologically normal adult has to be already pre-reflectively self-aware to be able to grasp one-self reflectively. This brings to mind the transformative rather than the additive views of rationality (McDowell, 1996; Boyle, 2012) in the analogical sense that human beings’ conceptual/reflective capacities transform their manner of being self-aware even pre-reflectively. And from an empirical psychological point of view, this would amount to the presence in us and not in other animals, of specific memory, executive, joint attentional, and language skills which *essentially* transform our self-conscious life and whose loss in brain damage renders a person completely unable of being self-aware. If that was the case then, when a patient with impaired awareness lacked the capacity to reflectively grasp oneself then this person would also lack the capacity to be pre-reflectively self-aware.

Additionally, it would be empirically useful to demonstrate that a person with impaired awareness can be self-aware even if he lacks higher-order reflective capacities. This information would potentially have important scientific repercussions because it could help embed the notion of minimal selfhood into the neuroscientific models of consciousness influencing any future experimental methodology. And information about the presence of minimal self-awareness in reflectively incapacitated patients will have at least some effect on these patients’ medical management and potentially ethical/legal implications about end-of-life decision making^9^.

## Discussion

With these matters in mind we now turn to the last section of the paper. It is composed of two subsections: A penultimate part where we take up our original title question to discuss the potential importance, the presence of PRSA in patients in MCS might have as empirical evidence, to experiential minimalism and a final part where we propose a way to test PRSA in such patients.

### Is the “Minimally Conscious State” Patient Minimally Self-Aware?

In a recent article in which he defends his position of experiential minimalism, the view that all our experiences are fundamentally characterized by a feeling^10^ of mineness Zahavi asks: “*If it is the case that our experiences are accompanied by a sense of self, is it then something that holds with necessity, such that it characterizes all experiences, however primitive or disordered they might be? Is it something that only holds for normal, adult, experiences? Or might it be something that only holds under rather special circumstances, say, when we reflectively scrutinize and appropriate our experiences*?” (Zahavi, 2017: p. 10) This is related to the view examined in previous paragraphs about bodily self-awareness as the type of minimal self-awareness which underscores awareness in general^11^.

But if we take into account what we have said above about DOC, are we allowed to additionally hold that PRSA characterizes all possible experiences, however “*primitive or disordered*,” as Zahavi asks? Or is it only for “*normal, adult, experiences*?”

In his discussion of psychopathology and depersonalization in schizophrenics, in the same article, Zahavi seems to argue that even in those extreme cases where one’s experiences are presented to him as not his own “*a dimension of self and self-consciousness remains*.” After all, these are experiences manifested in the first personal dimension, however impaired self-awareness therein might be and not in the second person as if one had to mind-read someone else’s thoughts and intentions (Ibid: 15).

But let’s ponder a little about the case of the newly diagnosed patient with MCS. This is someone who has previously been completely unresponsive (coma) and who has just acquired some primitive form of awareness. In the typical case, she merely tries to grasp your hand when you pinch her, unsuccessfully attempts to respond to your spoken urges to move a finger, sluggishly fixates on familiar faces or on her own when presented in the mirror or turning toward the sound of her own name. These are individuals who usually cannot communicate, do not show complex semantic understanding of objects of common usage presented to them, and who sometimes grasp but cannot manipulate those objects in a practically rational manner. We can, therefore, imagine the patient in MCS as someone who completely lacked awareness previously (and consequently self-awareness) and who is in the process of *becoming aware*. Again, the question is whether this primitive awareness that this patient awakens into (as can be judged by her behavioral responses or indirectly by neuroimaging or other means) involve a pre-reflective awareness of herself. We have seen that the current empirical studies and diagnostic tools are structured in such a manner as to evaluate *reflective* self-awareness as in own-face and own-name recognition. Being so they do not offer empirical evidence as to whether a PRSA is involved.

In distinction with schizophrenia where higher-order cognitive abilities are usually preserved, I believe the suggestion can be made that MCS represents *a limit case* the investigation of which could shed light on the question about what-it-is-like for someone who lacks higher-order conceptual abilities to be nevertheless aware. This would presumably be an occasion of pure self-awareness uncontaminated, so to say, by reflective acts, a chance to scientifically investigate the thin distinction between minimal self-awareness and reflectively laden self-awareness from pathology. Would this limit case of non-reflective self-awareness from pathology be a case of minimal self-awareness as Zahavi or others might suggest?

So to further narrow our investigation as to its theoretical aspect, I believe the inquiry about PRSA in MCS is vital in clarifying whether either or both of the following two proposals stand:

(1) That the self-awareness involved in having experiences is not an occurrence of an actual reflective grasping of those experiences and,
(2) That the self-awareness involved in having experiences does not require an overall ability to reflect.

The first proposal establishes in negative terms that self-awareness is primarily a pre-reflective awareness of the self as an embodied subject of experience and not an occasion of *actual* self-reflection. It refers to *actual* self-reflection as not being a necessary condition for self-awareness. Actual here meaning a reflective ability presently occurring in contradistinction to a reflective ability dormant at the time but with the potential, the capacity to occur. The second proposal, which to my knowledge is not specifically discussed as a topic in the relevant theoretical or empirical literature, is that this embodied subjectivity can figure in experience even in the limit case where one does not *absolutely* have the capacity to reflect or conceptualize as maybe the case is with young infants and a subgroup of patients in MCS without reflective abilities. It refers to the possession of PRSA even when there is *no potentiality* for self-reflection. It refers to the *potential* for self-reflection as not being a necessary condition for self-awareness^12^.

If we now flesh out the above two assumptions with the empirical issue we tackling here, the following two questions will constitute the final incarnation our inquiry takes:

• Does the patient in MCS possess PRSA even in the hypothetical case where he is utterly unable to reflect or
• Does he possess PRSA only in the case where he is at least able to practice self-reflection?

That is, should an awareness impaired patient, who does not exhibit overt or covert responses of reflective self-awareness, considered to be also PRSA possessing? Inversely, should he be considered possessing PRSA only in the case where he exhibits at least some minimal form of reflective capacities?

But another important task should also be attended to in consequence: How should the experimental setting be staged so that a pre-reflective aspect be disambiguated from a reflective aspect of self-awareness, in order to be able to test the above distinctive possibilities?

I will close this paper with some thoughts about what type of empirical evidence would be instrumental in responding to the above two questions.

### First-Person Perspective-Taking

As we saw, the notion of minimal self-awareness we examine in this paper primarily concerns the implicit and immediate awareness of our embodied subjectivity. A significant characteristic of this is the fact that in our perceptual engagement with our environment, we always have a tacit awareness of our body as the zero perspectival point of orientation in relation to the objects of our perceptual and practical interest. This embodied self of perception is experientially given in a pre-reflective manner whenever we direct our perceptual attention to the spatial objects around us. It is the awareness I have that I am observing from my absolute perspectival “*here*” the array of objects that are situated “*there*” in my peripersonal space^13^. And similarly, it is the sense of my embodied “*here*” when I reach and manipulate objects in motor actions.

**FIGURE 2:**
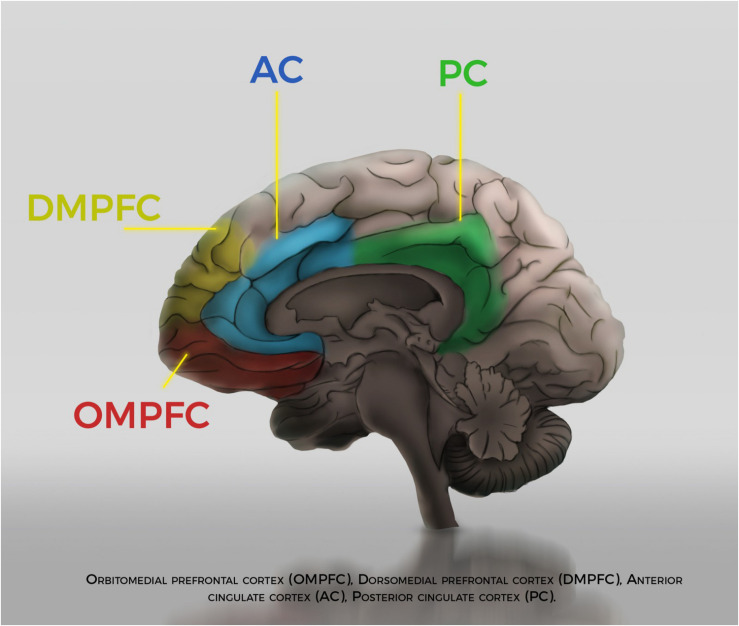
Artist’s representation of Cortical Midline Structures.

Now, if one browses through the currently published neuroscience of self-awareness, there has been empirical interest in recent years in the so-called Cortical Midline Structures (CMS), an extensive area of the medial aspect of the frontal and parietal lobes of cerebral hemispheres. These brain areas show elective and reproducible activity in neuroimaging studies with various self-referential tasks (Figure 2). But again, the majority of these tasks are self-referential in a reflective sense: reflection on one’s own personality traits, evaluation of self-referential statements, autobiographical memory tasks, etc. (review: Northoff and Bermpohl, 2004; Northoff et al., 2006; Frewen et al., 2020)^14^. These self-reflection tasks, once more, seem to be of limited use to our inquiry about what we might call *the neural correlates* of PRSA. If we consider the empirical aspect of our investigation here, what we need is to establish an experimental connection between some well-circumscribed aspect(s) of PRSA with a brain area or with an electrophysiological response.

In a 2004 fMRI study, researchers tested the taking of perceptual 1st Person Perspective (1PP) in 11 normal subjects as opposed to them taking the 3rd person perspective, in an attempt to detect the relevant neural correlates during these two psychological stances (Vogeley et al., 2004). To achieve this, they asked subjects to adopt either an egocentric spatial frame of reference by counting the number of red balls seen from their point of view or to adopt an allocentric frame of reference by counting the red balls from the perspective of another person (an avatar on a screen)^15^. Their assumption that these two mental acts were supported by two different neural processes paid off since different brain regions were consistently activated in each case. More specifically, to cling only on the information presented in that paper that is of interest to us, specific brain areas^16^ were consistently activated when taking the 1PP. At the same time, other brain areas showed deactivations^17^.

Without delving into neuroanatomical details here and whatever the definite relevant brain areas prove to be in future studies, I believe this empirical study constitutes an example of how to evaluate an aspect of minimal self-awareness through neuroimaging^18^. My suggestion would be: Since some brain regions show consistent activations when normal subjects take the 1PP, then subjects with impaired awareness who show minimal signs of awareness (as patients in MCS do) would show the same activations during similar tasks. Given an experimental paradigm could be developed so that 1PP could be assessed in MCS subjects and given some of those subjects exhibited activation of the relevant brain areas, we would be entitled to infer that those patients are minimally self-aware regardless of the fact that they might exhibit or not exhibit self-reflective abilities on additional testing^19^. This would be important to see because, on the one hand, it would suggest that these patients in MCS possess PRSA, and on the other, it could help us empirically discern whether this PRSA is present with or without the possession of additional reflective abilities^20^.

What if these patients exhibited 1PP activations only in the case they also exhibited self-reflective capacities when additionally tested? Wouldn’t that suggest that it is only when someone has the ability to self-reflect that one also possesses PRSA? And would that scenario then announce a blow to the *experiential minimalism* thesis since it would seem that it is only because self-reflective ability is already available as a potentiality that one is self-aware when one has experiences? That is, would experiential minimalism find experimental support only in the case where 1PP turns out to be detected even in the absence of self-reflective abilities? Or could experiential minimalism rather be compatible with the empirical proof of the presence in a subject of minimal self-awareness only when that subject possesses the capacity for self-reflection?

I will leave these important questions open for future discussion. I believe that if this paper has achieved anything was in making an effort to clarify the different notions of minimal awareness as discussed in these distinctive areas of research and practice and to elaborate on a possible way to establish a mutually illuminating link.

## Conclusion

Minimally Conscious State patients constitute a subgroup of awareness impaired patients who show minimal signs of awareness as opposed to VS patients who do not exhibit any such signs. While the empirical literature is rich in studies investigating either overt or covert signs of awareness in such patients, the question of self-awareness has only been scarcely addressed. Even in the occasion where self-awareness is evaluated, it is only higher-order or reflective self-awareness that is the target of such investigations. In the first part of this paper, I briefly reviewed the relevant clinical neuroscience literature to demonstrate that the conception of self-awareness at play in such studies is that of reflective self-awareness. According to this research area, patients in a MCS are minimally self-aware in the sense that they possess a quantitatively minimal capacity for reflective self-awareness. In the second part, I presented the philosophical notion of pre-reflective (or minimal) self-awareness. This was shown to primarily refer to the implicit awareness of our embodied subjectivity, which essentially permeates all our experiences. As discussed, this minimal self-awareness is not explicitly addressed when clinically or experimentally assessing patients in a MCS. My suggestion is that neuroimaging studies targeting minimal self-awareness as in First-Person Perspective-taking paradigms might be used with patients in MCS to shed light on the question of whether those individuals are minimally self-aware even in the case where they lack self-reflective abilities.

## Author Contributions

The author confirms being the sole contributor of this work and has approved it for publication.

## Conflict of Interest

The author declares that the research was conducted in the absence of any commercial or financial relationships that could be construed as a potential conflict of interest.

## Footnotes

1 I will be using the terms “pre-reflective self-awareness” and “minimal self-awareness” interchangeably. The content of these terms will be discussed in more detail later in the text.

2 http://www.tbims.org/combi/crs/CRS%20Syllabus.pdf.

3 To use a Gibsonian term common in the neuroscience of motor actions.

4 But why should we consider own face mirror tracking in such subjects as indicative of self-recognition, let alone as a higher order self-recognition at all? For Laureys et al. (2007: p. 725) “*the only clinical consciousness scale possibly referring to self-consciousness in DOC is the CRS-R. The CRS-R indeed explicitly tests patient’s visual fixation and tracking using a moving mirror*.” But since the CRS-R does not differentially test the patient for own vs. other face recognition we have to consider the possibility that the patient does not fixate on the mirror because he recognizes his own face as his own but merely because he recognizes somebody’s face looking back at him. This is a possibility that maybe future revisions of clinical tools assessing awareness impaired patients should consider. Nevertheless my assumption here is that the mirror test in CRS-R reflects a recognition by the subject of his own face, a capacity which is considered to be an example of higher order self-awareness by the relevant developmental psychology and philosophical literature.

5 It is of course no wonder that patients behaviorally diagnosed as in MCS perform better in these neuroimaging studies. The issue is that a number of behaviorally characterized VS patients also seem to have the capacity to actively perform imagination tasks. This is the reason that terms such as Cognitive Motor Dissociation (Schiff, 2015) and Functional Lock-in Syndrome (Formisano et al., 2013) were coined to refer to patients who seem to be conscious and capable of performing higher order cognitive acts and yet seem completely unresponsive.

6 “[S]tudies with healthy participants appear to show that reaction to one’s name is not automatic but can be involuntary […] it is accompanied with an explicit recognition of one’s name in healthy participants. However, this does not prove that the patients’ P300 brain response to their names reveals an explicit recognition. […]In everyday life one’s name is often used as an alerting external stimulus provoking an orienting response. Hence, the patients’ P300 response might merely reflect a conditioned orienting response due to hearing one’s name” (Laureys et al., 2007: p. 732).

7 But see also (Bagnato et al., 2015) where the sympathetic skin response to presented self-face images was studied as evidence of recognition of their own face in normal subjects and patients with DOC. There are also other diagnostic studies for awareness in DOCs which, for brevity purposes, I do not mention in the text for example PET, EMG, qEEG, eye tracking tests (review Bender et al., 2015, also Lehembre et al., 2012).

8 See also (Legrand, 2007; Nida-Rümelin, 2017).

9 Why would it be clinically important at all to prove that a patient possesses an awareness of himself as an embodied subject of his (however deprived) perceptual experience while at the same time proving that he is unable to perform reflective tasks? First and most importantly it would plainly demonstrate that he is conscious. This would entail that medically he needs to be managed as the conscious person he is, a subject that potentially feels pain and has emotions about his condition, even though he lacks the capacity to reflectively grasp them and express them. Secondly being pre-reflectively self-aware might prove to be a positive factor in these patients’ prognosis. And thirdly, if we take the ethical aspect into account he needs to be treated as a conscious human being (even if minimally) which would lead to specific legal decisions regarding his fate. Consequently, I do not think that the question about the possession of PRSA in the absence of reflective capacities should be taken lightly.

10 “Feeling” or “sense” with the meaning of “implicit awareness” or “what-it-it-liken-to-be” and not of sensation content (Zahavi, 2008: 117, 125, 145). See also (Kriegel, 2003).

11 See also Picolas and Soueltzis (2019) where the notion of PRSA as presented in phenomenological philosophy is discussed in relevance to the contemporary embodied subjectivity notions of “sense of ownership” and “sense of agency.”

12 The actuality/potentiality distinction above should be understood in its Aristotelian sense. This classical distinction is instrumental for the transformative view of rationality in philosophy of mind as mentioned in the text above (Boyle, 2012). In developmental psychology, this view finds empirical support in the work of Tomasello (1999, 2019). It would, of course, overwhelm the scope of this article if we delved into more details about this here.

13 This is reminiscent of the ecological self-according to Gibsonian psychology (Neisser, 1988).

14 A broader set of brain regions with a similar function termed the Default Mode Network (DMN) has also been proposed and studied in regards to DOCs patients (Crone et al., 2011; Fingelkurts et al., 2012). But see (Legrand and Ruby, 2009) which contest this view about the role of these structures as being self-referential at all and propose an alternative interpretation. See also (Grivaz et al., 2017).

15 The authors make the distinction additionally between 2PP and 3PP, that is that of another person’s view and the neutral Cartesian perspective view which I don’t discuss here so that I do not complicate my argument.

16 “[O]ur data clearly demonstrate differential brain activations associated with taking 3PP as opposed to 1PP. Whereas specific activations in the precuneus, the right superior parietal and right premotor cortex were found during 3PP, a differential increase of activation in mesial cortical regions was observed during 1PP” (Ibid 819).

17 A previous study had similar results: “Evidence from functional imaging, neuropsychology and lesion data imply medial cortical structures (comprising anterior medial prefrontal, medial parietal and posterior cingulate cortex) and inferior lateral parietal cortex as the basic neural mechanisms involved in 1PP” (see also Vogeley et al., 2001; Vogeley and Fink, 2003; Vogeley and Gallagher, 2011).

18 “The data suggest that in addition to joint neural mechanisms […] 3PP and 1PP rely on differential neural processes. Mesial cortical areas are involved in decisional processes when the spatial task is solved from one’s own viewpoint, whereas egocentric operations from another person’s perspective differentially draw upon cortical areas known to be involved in spatial cognition.” (Vogeley et al., 2004: p. 817).
