# Corrigendum: Is the “Minimally Conscious State” Patient Minimally Self-Aware?

**DOI:** 10.3389/fpsyg.2020.628618

**Published:** 2020-12-01

**Authors:** Constantinos Picolas

**Affiliations:** ^1^Department of Philosophy, University of Patras, Patras, Greece; ^2^Department of Neurosurgery, Nicosia General Hospital, Strovolos, Cyprus

**Keywords:** self-awareness, minimally conscious state, vegetative state, pre-reflective self-awareness, experiential minimalism

In the published article, there were errors in affiliations 1 and 2. For affiliation 1, instead of “Department of Philosophy, University of Patras, Strovolos, Greece,” it should be “Department of Philosophy, University of Patras, Patras, Greece.” For affiliation 2, instead of “Department of Neurosurgery, Nicosia Genera Hospital, Patras, Cyprus,” it should be “Department of Neurosurgery, Nicosia General Hospital, Strovolos, Cyprus.”

The authors apologize for these errors and state that they do not change the scientific conclusions of the article in any way. The original article has been updated.

